# Case Report: Five Adult Cases of H3K27-Altered Diffuse Midline Glioma in the Spinal Cord

**DOI:** 10.3389/fonc.2021.701113

**Published:** 2021-12-08

**Authors:** Quanquan Gu, Yajing Huang, Hao Zhang, Biao Jiang

**Affiliations:** ^1^ Department of Radiology, Second Affiliated Hospital, School of Medicine, Zhejiang University, Hangzhou, China; ^2^ Department of Pathology, Second Affiliated Hospital, School of Medicine, Zhejiang University, Hangzhou, China; ^3^ Department of Medicine, Faculty of Medicine & Dentistry, University of Alberta, Edmonton, AB, Canada

**Keywords:** diffuse midline glioma (DMG), radiology, CNS neoplasm, spinal cord, pediatric-type gliomas

## Abstract

**Background:**

Diffuse midline glioma with H3K27-altered (DMG-H3K27a) is a novel tumor entity of the pediatric-type diffuse high-grade tumor in the latest WHO CNS 5. It mostly affects children and is only rarely found in adults. The tumor has a high level of aggressiveness, with a rapid progression and bad prognosis. In adults, the spinal cord is the most common site of DMG-H3K27a. Rare adult cases of primary DMG-H3K27a in the spinal cord were reported in this study, together with clinico-histopathologico-radiographic data.

**Methods:**

From January 2016 to December 2020, we conducted a retrospective study of five adults with primary DMG-H3K27a in the spinal cord, analyzing their clinical, pathohistological, and radiographic datasets from the first diagnosis to follow-up.

**Results:**

All five patients were diagnosed for the first time and were given full treatment. In three of the five patients, post-operative follow-up revealed tumor recurrence. The longest survival of the five patients was 45 months at the time of report submission, while the longest progression-free survival (PFS) following surgery was 20 months. Immunohistochemical studies showed the tumors featured aggressive behavior (grade 4) and were positive for the H3K27M mutation. The radiographic appearances were varied, but they were all initially mistaken as benign. DMG-H3K27a in the spinal cord was characterized by isointense/hyperintense on T1WI and isointense/hyperintense on T2WI, as well as cystic necrosis and peripheral spinal cord edema, as well as central canal enlargement and other types of enhancement.

**Conclusion:**

This is the first case report focusing on adult DMG-H3K27a of the pediatric-type diffuses high-grade gliomas in the spinal cord. In our cases, we discovered the following: 1) adults had a better prognosis with a longer PFS compared with prior pediatric reports; 2) despite aggressive behavior under the microscope, radiographic appearances of the tumors were less aggressive; and 3) adjuvant treatment, including TCM, may have played a role in the prognosis.

## Introduction

Diffuse midline glioma with H3K27-altered (DMG-H3K27a) is a newly recognized pediatric-type diffuse high-grade tumor entity in the fifth edition of WHO Classification of Tumors of the Central Nervous System (CNS5, 2021) (1). It occurs primarily in children, with adults being affected occasionally (1). Indeed, DMG-H3K27a had been characterized as diffuse midline glioma with H3K27M mutations (DMG-H3K27m) in the 2016 WHO classification (2). Its nomenclature has recently been revised in recognition of other altered mechanisms (e.g., *EZHIP* protein overexpression) that could underlie the pathogenesis of tumors (1). Clinically, the diagnostic criteria for DMG-H3K27a consist of a diffuse growing pattern (i.e., infiltrating), a midline anatomic location (including the thalamus, cerebellum, brain stem, and spinal cord), and H3K27-specific neuroglial mutations (2, 3), in addition to supporting histopathological and molecular evidence (4).

It is of note that DMG-H3K27a appears highly heterogeneous in terms of pathology and imaging characteristics, and the prognosis for such malignancy also differs depending on the affected population. While DMG-H3K27a was formerly considered a rare high-grade glioma in children with a limited survival cycle, it can also affect adults. However, unlike in children, tumors in adults tend to occur in the spinal cord rather than the pons (5). To date, adult neoplasm has been reported in just a few cases, thereby greatly limiting the elucidation and generalization of adult DMG-H3K27a involving the spinal cord (6, 7). This report describes the clinico-histopathologico-radiographic features of five adult patients carrying pathology-confirmed DMG-H3K27 alteration in detail and presents a brief review of current literature relevant to the topic for future reference.

## Methods

Adult patients with DMG-H3K27a were retrospectively identified and recruited between January 2016 and December 2020 at Second Affiliated Hospital of Zhejiang University, School of Medicine, Hangzhou, China. The patients were limited to those who were diagnosed pathologically following prior brain surgeries, which was in line with the latest WHO-CNS5 guideline (1). Our study was approved by the institutional review committee and human research ethics board. Informed consent and/or assent was obtained from research subjects (or next of kin) prior to enrollment.

The clinico-histopathologico-radiographic profiles of the five adult cases with lesions in the spinal cord have been thoroughly described. Briefly, we performed a preoperative 3.0T MRI scanning on all the patients. Two experienced radiologists were in charge of interpreting the radiographic findings. Individuals with intracerebral tumor foci were excluded from the study. Patients’ past surgical procedures and postoperative therapies were well documented (see the **Supplementary Material** of this article). We also tracked prognostic factors such as tumor remission, recurrence, and progression. Among them, the modified Response Assessment in Neuro-Oncology criteria (8) were adopted to define progression, which may or may not be accompanied by worsened clinical symptoms, requiring treatment adjustment. The duration (months) between the start of treatment and the first occurrence of tumor progression, relapse, or death (from any cause) was referred to as progression-free survival (PFS) (8). For those without disease worsening, the PFS endpoint was set at the date of their last follow-up visit. Recurrence was confirmed using a combination of self-perceived new or exacerbated clinical manifestations and definite radiographic progression.

## Case Description

### Demographic Information

We studied 18 adult DMG-H3K27a patients, including 5 cases arising in the spinal cord, 5 cases in the thalamus, 3 cases in the brainstem, 3 cases in the basal ganglia, and 2 multifocal cases (basal ganglia-thalamus-brainstem and frontotemporal insula-thalamus-brainstem). The clinical profiles of the five patients with spinal cord lesions are summarized in **Table 1**. Their chief complaints lacked specificity and were mostly about symptoms connected to the spinal cord portions where the lesions were found. Patients’ ages ranged from 27 to 65 years old at initial diagnosis of DMG-H3K27a (previously known as DMG-H3K27m), with a mean age of 42.4-year-old. No significant gender bias arose, and two male and three female patients were included within the study cohort.

**Table 1:** Clinical information.

No	Age/Sex	Chief complaint	PE	Preoperative diagnosis	Post-surgery PFS (month)
1	49/M	Paroxysmal waist soreness for 3 months	LEMS (L/R) grade 2/3, hypesthesia in the right limb below-the-knee, numbness in the bottom of left foot	Schwannoma	4
2	39/F	Back pain for 11 days, numbness in both lower limbs, and urination defecation difficulties for 1+ week	numbness below the nipple- level	Astrocytoma	12
3	32/F	Numbness in left limbs for 1+ months	LEMS grade 5- (L), numbness and hypesthesia below T 2 level	Ependymoma/ Schwannoma	7.5
4	65/M	Paroxysmal and progressive weakness and numbness in the right lower limb, particularly at night	Negative	Astrocytoma	9
5	27/F	Numbness in both toes for 2+ months and back pain for 10+ days, relieving when side lying	Negative	Ependymoma	20

LEMS, lower-limb muscle strength; PFS, Progression free survival.

### Treatment

All of these patients were treated surgically, and the surgical procedures included completely total (**case 2**), near-total (**case 4**), and subtotal (**case 1, 3, 5**) tumor resections, respectively. All were given postoperative chemoradiotherapies, along with symptomatic treatment, physiotherapy rehabilitation, and Traditional Chinese Medicine (TCM), as per the National Comprehensive Cancer Network (NCCN) recommendations. Some of the patients received additional immune-targeted therapy (**case 3**) and/or subsequent surgeries (**case 1, 3, 5**). It is also worth noting that all five patients have been prescribed adjuvant therapies, namely traditional Chinese acupuncture, moxibustion, and herbal medications. The treatment information is further described in the **Supplementary Material**.

Three patients (**cases 1, 3, 5**) had experienced tumor recurrences. The patients included in this report survived for 10 to 45 months after surgery, with a PFS ranging from 4 to 20 months. Specifically, **case 1** was a relapsed patient who deteriorated after 14 months of adjuvant chemoradiotherapies following the initial subtotal tumor resection. Another subtotal tumor resection and radioactive particle implantation were then administered to treat this patient. **Case 3** had a PFS of 7.5 months since the first near-total resection with following chemoradiotherapies, during which the patient received radioactive particle implantation 5 months later. The lesion spread to intracranial lateral ventricular walls and the thoracic medullary segment 11 months later after the aforementioned interventions, leading to a second-time subtotal tumor resection of the thoracic medullary lesion combined with targeted chemoradiotherapies. **Case 5** was a patient who experienced a relapse 20 months after the initial subtotal excision of the tumor. By that time, the patient underwent another substantial resection of the lesion, but three months later, the patient developed a second relapse with, the tumor spread to the cervical medullary section. Importantly, both **cases 2 and 4** received total tumor excision complemented by additional adjuvant therapy, and neither demonstrated relapses as of the submission date.

### Histopathological Presentation

**Figure 1:**
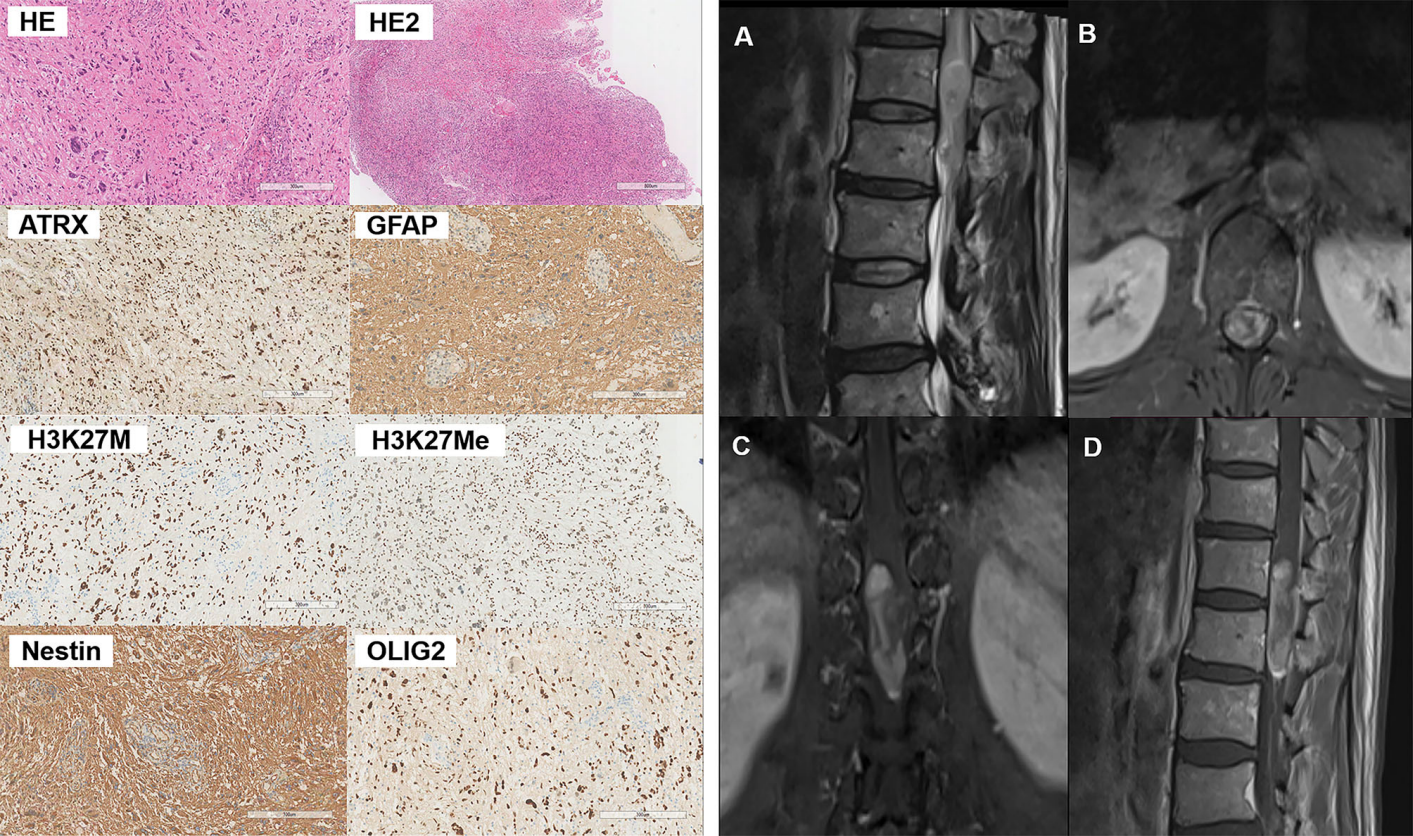
Pathological and radiographical manifestations of the Case 4 patient. Left: Results of HE and immunohistochemical staining of the tumor tissues. Right: MRI images of the tumor that was located at the T12-L1 level. The tumor exhibited heterogenous iso-hyperintense with speckled hyperintense spots inside on T2WI **(A)** and patchy and cystic with nodules enhancement **(B–D)** on contrast-enhanced MR images.

The histopathological and immunohistochemical analyses were performed for all 5 cases. At high magnification, all high-grade gliomas with heteromorphic glial cells were shown and the immunohistochemical results were presented in **Table 2**. Histologically, the gradings of these enrolled patients were all classified as WHO grade 4. All five cases were subjected to histological and immunohistochemical examinations. The morphologic differentiation of the five patients was astrocytic glioblastoma, with microvascular proliferation and cytological features of significant nuclear atypia, pathological mitosis, and cytologic anaplasia. Three out of the five patients (**cases 3, 4, and 5**) had necrosis, and 1 patient (**case 1**) demonstrated sparse fibers and myxoid alteration. Immunohistological tests revealed that all the five cases were positive for H3K27M mutations with an absence of H3K27Me3 expression. In addition, our cases had immunohistochemistry positive levels of the proliferative index Ki-67 ranging from 5% to 30%, with one instance (**case 1**) exhibiting sparse fibers and myxoid tumor cells at the highest Ki-67 level of 30%. To illustrate, the histological and immunohistochemical findings of **case 4** are shown in **Figure 1**.

**Table 2:** Immunohistochemical findings.

No	Age/Sex	WHO grade	ATRX	GFAP	Syn	H3K27M	H3K27Me3	Nestin	OLIG2	P53	Ki-67	Other features
1	49/M	4	Retain	+	+	+	Partly loss	+	+	–	30%+	Sparse fibers and myxoid change
2	39/F	4	Retain	+	+	+	Partly loss	+	+	–	15%+	–
3	32/F	4	Retain	+	NA	+	–	+	+	scantly +	20%+	Necrosis
4	65/M	4	Retain	+	focally +	+	Partly loss	+	+	–	5%+	Necrosis
5	27/F	4	Retain	+	+	+	NA	+	+	scantly +	5%+	Necrosis

NA, not applicable.

"-" means negative expression; and "+" means positive expression.

### MRI Presentation

All the five patients included in this study had malignancies that were intramedullary. Four (**case 1, 2, 3, 4**)? of the five cases had lesion located in the thoracic segment, whereas only one (**case 3**)? had a lesion located in the cervical segment. The tumors all grew along the long axis of the spinal cord, with the longest segment distribution reaching three vertebral body heights (**case 1, 4, 5**). Prior to initial surgery, none of the patients had any intracranial abnormalities. The T1 and T2 signals on plain MRI scans displayed a variety of expressions for all the cases, as did the T1 enhancement signals. All lesions exhibited diverse enhancement, therefore we used descriptions from existing research to characterize the enhancement patterns as patchy, ring-like, homogenous, and cystic with nodules based on their appearance (9). The MR image description of each case is summarized in **Table 3**. The MRI presentations of the cervical spinal cord lesions (**case 4**) are illustrated in **Figure 1**.

**Table 3:** MRI presentations.

No	Anatomical location	TumorBoundary	Necrosis and cysts	Rim of low T2 signal intensity	Marrow edema	T1WI	T2WI	Enhancement pattern*
1	T10-12	Clear	+	–	–	Isointense	Iso- or hyperintense	Patchy
2	T1-2	Clear	+	–	+	Hypo- or isointense	Iso- or hyperintense	Ring-like and cystic with nodules
3	C1-2	Clear	+	+	+	Isointense	Hyperintense, with spinal cord swelling and the enlarged central canal	Patchy, ring-like and cystic with nodules
4	T12-L1	Obscure	–	–	+	Iso- or hyperintense	Heterogeneous iso- or hyperintense, with speckled hyperintense spots inside	Patchy and cystic with nodules
5	T4-6	Clear	+	–	+	heterogeneous hypointense	heterogeneous hyperintense, with spinal cord swelling and enlarged central canal	Patchy

*Enhancement pattern: patchy, homogeneous, and cystic with nodules in appearance (9).

"-" means negative expression; and "+" means positive expression.

## Discussion

DMG-H3K27a was recently introduced to the categorization of the pediatric-type high-grade diffuse gliomas in the latest WHO-CNS5, which divided diffuse gliomas into adult and pediatric types (10). The modified nomenclature reflects the multiple mechanisms by which the pathogenic pathway can be altered in these tumors (e.g., EZHIP protein overexpression) (1). This novel tumor entity was previously recognized as DMG-H3K27m (2) and diffuse intrinsic pontine gliomas (DIPGs) (11). Adults with pediatric-type DMG-H3K27a in the spinal cord are still rare, thus the demographic and medical profiles of the newly defined tumor have yet to be studied. To the best of our knowledge, this is the first report of adult cases with primary DMG-H3K27a in the spinal cord.

From January 2016 to December 2020, we respectively collected 5 adult patients who had primary DMG-H3K27a but were free of intracranial foci or other neoplasms. Three patients (**case 1, 3, 5**) experienced recurrence during the follow-up, with a PFS of 14, 7.5, and 20 months, respectively. The 5 patients have survived for 10 to 45 months as of the submission date, with the longest PFS reaching 20 months following the initial surgery. The histological features of the tumors showed aggressive behavior, with microvascular proliferation and cytological abnormalities such as substantial nuclear atypia, pathological mitosis, and cytologic anaplasia in all five cases. In the meantime, necrosis, sparse fibers, and myxoid alteration were also found in some of these cases (see **Table 2**). Although the radiographic manifestations of these tumors differed from case to case, they were all initially misdiagnosed as low-grade intramedullary neoplasms.

Amongst all the 18 cases with H3K27M mutations, the preferred sites for adult DMG-H3K27a were the spinal cord and thalamus, which were consistent with findings from prior literature (5, 9, 11, 12). Besides, adult DMG-H3K27a has been rarely found in the third ventricle, pineal region, hypothalamus, basal ganglia, corpus callosum, or cerebellum (5, 13). The histological grading of all five patients in the present report was WHO grade 4 with tumor cells behaving aggressively. All of these gliomas have heteromorphic glial cells with microvascular proliferation and cytological hallmarks of substantial nuclear atypia, pathological mitosis, and cytologic anaplasia, which can be detected at high magnification (see **Table 2**). In addition, necrosis, sparse fibers, and myxoid alteration were seen in a subgroup of the patients. Astrocytoma is the most prevalent DMG-H3K27m morphologic variant, followed by oligodendrocytes, angioblasts, and neuropil-like structures, such as hairy cell myxoid, ependymoid, sarcomatoid alterations, and so on (5, 14). In a previous study, only 15 out of 25 patients with spinal tumors harboring H3K27 mutations were histologically graded 4 (15). Given the small sample size in our study, extensive datasets would be warranted to confirm the greater incidence of high-grade gliomas that we discovered. DMG-H3K27m, the most influential member of the DMG-H3K27a family, is characterized by a K27M mutation in either H3F3A or HIST1H3B/C, which causes a drop in their methylation levels affecting gene transcription and leading to cancer progression (16). Histologically, the tumor infiltrates grey and white matter structures. Mitosis exists in most cases, but it is not necessary for diagnosis. Microvascular proliferation and necrosis can also be seen in DMG-H3K27m. Although the histological presentations of the tumor span a wide range across low to high grades, it has been classified as grade 4 based on its highly malignant biological behavior characterized as a progressive and diffusive growth pattern. As for immunotyping, DMG-H3K27m is positive for CD56, S100 and OLIG2, whereas immunoreactivity to GFAP remains variable. In parallel, synaptophysin immunoreactivity can be focal, while chromogranin-A and NeuN are not typically expressed (17). Loss of nuclear ATRX expression exists in about 10-15% of cases. Nuclear p53 immunopositivity exists in about 50% of cases. It has been identified that recurrent heterozygous mutations are at position K27 in the histone coding genes H3F3A, HIST1H3B, and HIST1H3C in high-grade gliomas from the pons, thalamus, and spinal cord (18, 19). High-level focal amplifications included PDGFRA, MYC/MYCN, CDK4/6, CCND1-3, ID2, and MET. Homozygous deletion of CDKN2A/B or loss of RB1 or NF1 can only be detected in very rare cases (20).

MRI can reveal not only fine structural features but also tissue components. Preclinical and clinical assessments, as well as follow-up MRI examinations, were performed on the enrolled patients. DMG-H3K27m are naturally heterogeneous as indicated in the pathology section and as high-grade malignant tumors, the tumor composition and percentage of the components are complicated and flexible. Previous work has rarely addressed the radiographic presentation of the spinal cord DMG-H3K27m in adults (4), thus it still lacks comprehensive overview of the radiographic manifestations to the best of our knowledge. Except for one case at the cervical level (**case 3**), all the five intramedullary spinal tumors were at the thoracic level (**cases 1, 2, 4, and 5**). Although the cervical cord has long been assumed to be the most common site of intramedullary DMG-H3K27m (21), the predisposition to other regions such as the thoracic medulla has also been documented in both adults and children (4). We also discovered that the lesions were long-segmental, and most of the cases had a longitudinal axis length of three vertebral body heights, which was consistent with earlier research (9). DMG-H3K27a tumors have a wide range of MRI signal characteristics and enhancing patterns. FLAIR hyperintense, heterogeneous enhancement, and subependymal dissemination can be observed in pediatric MRI images (6), but in adults, the signals are homogeneous on plain MRI images with varied enhancement patterns (9). To characterize the enhancing lesions of the recruited patients, we employed a categorization mentioned in prior literature and defined them as patchy, homogenous, and cystic with nodules in appearance (9). On diffusion weighted images (DWI) the foci can sometimes have erratic enhancement patterns, although the majority of them were solid and mild signals (9). We were unable to offer DWI data because DWI scans were not done on any of our enrolled patients. It was worth noting, however, that the initial MRI presentation of the five patients all resembled a low-grade CNS tumor, which easily led to being misdiagnosed as benign tumors at the initial diagnosis, potentially delaying treatment. In case of a 25-year-old women (22) and a 28-year-old man (23), the DMG-H3K27m tumors were located in the cervical medulla, mimicking the manifestations of meningioma and ganglioneuroma on the images respectively. Other studies have suggested that patients with suspected gliomas at midline sites on MR should first undergo immunohistochemistry testing for H3K27M mutations, especially if tissues were difficult to collect and malignancy is difficult to diagnose based on histological examination alone (13). Our radiographic findings added to the evidence that DMG-H3K27a was more likely to have a benign-like manifestation at the first diagnosis.

The post-operative chemotherapy or radiation regimens of the five patients were also tracked and their PFS periods were documented (please refer to the **Supplementary Material**). Reaggravated symptoms and aberrant enhancements on imaging were used to diagnose the recurrence. The clinical features of the new tumors in the three patients who had relapsed were identical with those in the initial sites, confirming the diagnosis of tumor recurrence. The longest survival of the 5 patients had reached 45 months at the time of being reported, while the PFS after surgery had reached 20 months. Patients with H3K27 alterations are generally associated with a poor prognosis in children compared with wild-type individuals, with a two-year survival rate of less than 10% despite extensive therapy (24) and an additional two years minus survival compared with other high-grade gliomas occurring in children (21). However, it is becoming obvious that the H3K27 alterations might have a positive impact on adult patients. Specifically, Shreck et al. (13) have revealed that adult patients with diffuse midline gliomas tend to live longer than those with wild-type ones in the presence of H3K27M mutations (17.6 months vs. 7.7 months). When it comes to neoplasms located in the spinal cord, the adult survival rate is further improved, with a maximum survival of approximately 20 months (12, 15). According to the above findings, adult DMG-H3K27a patients are more likely to have a better prognosis than children, which could aid doctors in devising a more effective treatment plan.

It is worth mentioning that in addition to routine chemotherapy or radiation and supportive rehabilitation, TCM therapy may have played an important role in the relatively better prognoses of these five adult cases. TCM therapy, including acupuncture, moxibustion treatment, and herbal medicine, has long been used as an adjuvant solution for patients with malignant neoplasms in China, which can relieve symptoms as well as improve life quality. We believed that the joint effects of multiple measures as mentioned facilitated the longer PFS for DMG-H3K27a patients.

Given the low incidence of DMG-H3K27a in adults, future research needs to introduce a larger sample size that comprises more pathogenetic subtypes of the tumor family and more informative laboratory biomarkers and multimodal radiographical datasets, which would render more comprehensive and profound insights into adult DMG-H3K27a in the spinal cord.

## Conclusion

In conclusion, this is the first case report to focus on adult DMG-H3K27a of pediatric-type diffuses high-grade gliomas in the spinal cord from a clinico-histopathologico-radiographical aspect. Although under the microscope the tumors had aggressive behavior, their radiographical appearances were more like low-grade tumors, and the prognosis was more favorable than expected. Lastly, when encountering adult patients with suspected glioma arising at midline sites, an immunohistochemical examination for H3K27 alterations should be applied despite the seemingly less aggressive radiographical manifestations. The prolonged survival time could be the result of the comprehensive treatment according to NCCN recommendations and adjuvant TCM therapy.

## Data Availability Statement

The raw data supporting the conclusions of this article will be made available by the authors, without undue reservation.

## Ethics Statement

The studies involving human participants were reviewed and approved by Second Affiliated Hospital of Zhejiang University School of Medicine Ethics Committee. The patients/participants provided their written informed consent to participate in this study.

## Author Contributions

QG took the lead in writing the manuscript and provided MRI images. YH provided histopathological information and interpretation. HZ aided in manuscript writing and the acquisition of supplementary data. BJ was responsible for drafting and reviewing the manuscript and provided supervision. All authors contributed to the article and approved the submitted version.

## Funding

This work was supported by the National Natural Science Foundation of China (Grant No. 81701647).

## Conflict of Interest

The authors declare that the research was conducted in the absence of any commercial or financial relationships that could be construed as a potential conflict of interest.

## Publisher’s Note

All claims expressed in this article are solely those of the authors and do not necessarily represent those of their affiliated organizations, or those of the publisher, the editors and the reviewers. Any product that may be evaluated in this article, or claim that may be made by its manufacturer, is not guaranteed or endorsed by the publisher.
