# Supplementary material for: Case Report: Five Adult Cases of H3K27-Altered Diffuse Midline Glioma in the Spinal Cord
Source: Front Oncol. 2021 Dec 8;11:701113. doi: 10.3389/fonc.2021.701113 (PMC8694136; doi:10.3389/fonc.2021.701113)
Supplement: Supplementary file 1 [file DataSheet_1.docx]

**Supplementary Material**

**Treatment of all the reported cases following surgeries**

**Case 1: 49y/male**

The patient’s intramedullary spinal cord neoplasm was initially treated with subtotal tumor resection on November 12, 2018. The tumor was located in the ventral part of the T9-12 medulla and was 8.0cm*1.0cm*1.5cm in size, with no evident distinction between the tumor and normal medulla. After surgery, the patients had received Temozolomide chemotherapy and adjuvant radiation regimens 25 times (December, 2018-January, 2020) in the local hospital, where the chemoradiotherapies and other supportive treatments were administered in accordance with NCCN recommendations. In parallel, the patient received Traditional Chinese Medicine (TCM) treatments, namely scalp acupuncture and herbal therapy. However, the patient underwent a subtotal tumor resection combined with radiative particle implantation at the T10-12 level (January 28, 2020), following a prior recurrence of the tumor earlier in the month. The patient’s most recent follow-up appointment with our outpatient clinic occurred in February of 2020.

**Case 2: 49y/female**

The patient received a gross total tumor resection (GTR) on December 16, 2019. The intramedullary tumor had a size of 1.0cm*1.3cm*1.5cm at the T1-3 level with an indistinguishable margin. The patients received NCCN-guided postoperative chemoradiotherapies, combined with supportive treatments including symptomatic medications, physiotherapy rehabilitation and TCM at the local hospital. No further details were available. The patient was instructed to follow up with the neurosurgical outpatient clinic monthly for the first three months after the operation, and then every six months and onwards. No subsequent incidents were reported as of her most recent outpatient visit in January 2021.

**Case 3: 32y/female**

The patient received a subtotal tumor resection at the C1-2 level on September 28, 2018. She was subsequently administered chemoradiotherapies as per the NCCN guidelines in our ward. Specifically, she was scheduled to receive 40 gray per 20 fractions (40Gy/20F) radiotherapy twice, Temozolomide chemotherapy six times, and Carboplatin-Etoposide combined chemotherapy three times. In addition, we gave the patient intrathecal methotrexate and dexamethasone. During her hospitalization, supportive care and TCM medications were also prescribed. However, 7.5 months after the first surgery, the patient developed a tumor recurrence with thoracic medullary metastases in April 2019. As a result, another subtotal tumor excision coupled with the implantation of radioactive particle were conducted on September 18, 2019. Of note, the patient declined to take regular adjuvant therapy after the second surgery. In August 2020, the patient’s tumor recurred again, and was removed completely on August 3, 2020. Although adjuvant therapy was provided after this surgery, the MRI-confirmed intracerebroventricular metastases developed quickly. The patient was then given Temozolomide and Bevacizumab-targeted chemotherapy seven times, with intrathecal injections of cytarabine, methotrexate, and dexamethasone in between. Her latest follow-up visit took place on December 30, 2020.

**Case 4: 65y/male**

The patient had near-total tumor resection at the T12-L1 level, and the tumor size was 0.5cm*2.0cm*7.0cm, with no clear distinction between the tumor and neighboring medulla. The patient took postoperative treatments in the local hospital. Similarly, the adjuvant therapy comprised of chemoradiotherapies and other supportive regimens (i.e., TCM treatments and herbal remedies) based on NCCN suggestions, with specific details unknown. There was no tumor recurrence reported during his last follow-up visit on April 28, 2020.

**Case 5: 27y/female**

The patient underwent a subtotal tumor resection at the T4-6 level on March 28, 2017 at the local hospital. The tumor was 8.0cm*1.5cm*1.5cm in size with an infiltrating growth pattern. The patient was given Temozolomide chemotherapy regimen following the surgery, but the tumor recurred 20 months later (November 27, 2018) with diagnostic confirmation by MRI findings. The second subtotal resection was performed on December 5, 2018 followed by postoperative adjuvant treatments. However, the tumor again recurred 3 months after the second surgery. Guideline-recommended supportive treatments were administered afterwards. Her last follow-up visit was on May 28, 2019.
